# Big data from electronic health records for early and late translational cardiovascular research: challenges and potential

**DOI:** 10.1093/eurheartj/ehx487

**Published:** 2017-08-29

**Authors:** Harry Hemingway, Folkert W Asselbergs, John Danesh, Richard Dobson, Nikolaos Maniadakis, Aldo Maggioni, Ghislaine J M van Thiel, Maureen Cronin, Gunnar Brobert, Panos Vardas, Stefan D Anker, Diederick E Grobbee, Spiros Denaxas, Innovative Medicines Initiative 2nd programme, Big Data for Better Outcomes, BigData@Heart Consortium of 20 academic and industry partners including ESC

**Affiliations:** 1 Research Department of Clinical Epidemiology, The Farr Institute of Health Informatics Research, University College London, 222 Euston Road, London NW1 2DA, UK; 2 The National Institute for Health Research, Biomedical Research Centre, University College London Hospitals NHS Foundation Trust, University College London, 222 Euston Road, London NW1 2DA, UK; 3 Department of Cardiology, University Medical Center Utrecht, Heidelberglaan 100, Utrecht 3584 CX, The Netherlands; 4 MRC/BHF Cardiovascular Epidemiology Unit, Department of Public Health and Primary Care, University of Cambridge, Worts Causeway, Cambridge CB1 8RN, UK; 5 NIHR Biomedical Research Centre for Mental Health (IOP), King‘s College London, De Crespigny Park, London SE5 8AF, UK; 6 European Society of Cardiology (ESC), 2035 Route des Colles, Les Templiers - CS 80179 Biot, 06903 Sophia Antipolis, France; 7 Vifor Pharma Ltd, lughofstrasse 61, 8152 Glattbrugg, Zurich, Switzerland; 8 Department of Epidemiology, Bayer Pharma AG, Müllerstrasse 178, 13353 Berlin, Germany; 9 Division of Cardiology and Metabolism—Heart Failure, Cachexia & Sarcopenia; Department of Cardiology (CVK), Berlin-Brandenburg Center for Regenerative Therapies (BCRT), Charité University Medicine, Charitépl. 1, 10117 Berlin, Germany; 10 Department of Cardiology and Pneumology, University Medicine Göttingen (UMG), Robert-Koch-Strasse 40, 37099, Göttingen, Germany; 11 Julius Centre for Health Sciences and Primary Care, University Medical Center Utrecht, Heidelberglaan 100, 3584 CX Utrecht, The Netherlands

**Keywords:** Electronic health records, Health informatics, Bio-informatics, e-Health, Precision medicine, Translational research

## Abstract

**Aims:**

Cohorts of millions of people's health records, whole genome sequencing, imaging, sensor, societal and publicly available data present a rapidly expanding digital trace of health. We aimed to critically review, for the first time, the challenges and potential of big data across early and late stages of translational cardiovascular disease research.

**Methods and results:**

We sought exemplars based on literature reviews and expertise across the BigData@Heart Consortium. We identified formidable challenges including: data quality, knowing what data exist, the legal and ethical framework for their use, data sharing, building and maintaining public trust, developing standards for defining disease, developing tools for scalable, replicable science and equipping the clinical and scientific work force with new inter-disciplinary skills. Opportunities claimed for big health record data include: richer profiles of health and disease from birth to death and from the molecular to the societal scale; accelerated understanding of disease causation and progression, discovery of new mechanisms and treatment-relevant disease sub-phenotypes, understanding health and diseases in whole populations and whole health systems and returning actionable feedback loops to improve (and potentially disrupt) existing models of research and care, with greater efficiency. In early translational research we identified exemplars including: discovery of fundamental biological processes e.g. linking exome sequences to lifelong electronic health records (EHR) (e.g. human knockout experiments); drug development: genomic approaches to drug target validation; precision medicine: e.g. DNA integrated into hospital EHR for pre-emptive pharmacogenomics. In late translational research we identified exemplars including: learning health systems with outcome trials integrated into clinical care; citizen driven health with 24/7 multi-parameter patient monitoring to improve outcomes and population-based linkages of multiple EHR sources for higher resolution clinical epidemiology and public health.

**Conclusion:**

High volumes of inherently diverse (‘big’) EHR data are beginning to disrupt the nature of cardiovascular research and care. Such big data have the potential to improve our understanding of disease causation and classification relevant for early translation and to contribute actionable analytics to improve health and healthcare.

## Introduction

Electronic records relevant to the understanding of health and disease are found in diverse sources including not only the formal electronic health records (EHR) used in a growing number of healthcare organizations but also in omic, imaging, wearable and other data. These record data are increasingly being used for research, beyond the primary purpose for which they were collected. ‘A new era of data-based and more precise medical treatment’^1^ is envisaged in which the practice of medicine becomes ‘evidence generating’.^2^ One emerging prospect is the use of big record data to traverse the translational pathways from early discovery phases of translation to later implementation phases. Previous reviews on mining EHR have not had a focus on cardiovascular disease^3^ or have focused on cardiovascular care^4^^,^^5^ without a consideration of the translational pathways. We provide, for the first time, a critical review of big health record data for cardiovascular disease research across the translational spectrum, including early phases of discovery science, drug development and repurposing, and precision medicine, and later translational phases of learning health care systems, real world evidence, citizen-centred, and public health.

We review four areas in relation to big health record data:

(i) What data *resources* exist for cardiovascular disease research?
(ii) What are the *challenges* and *barriers* to realizing these opportunities?
(iii) What is the potential of such data in *early translational* research including discovery science, drug development and repurposing, precision medicine?
(iv) What is the potential of such data in *late translational* research including learning health care systems, real world evidence, citizen-centred and public health?

### Big health record data resources

‘Big data’ are usefully characterized by ‘variety, volume, velocity, and value’ (a fifth V, veracity, relating to data quality is dealt with below in the challenges section). EHR are intrinsically ‘big’ due to their complexity (‘variety’) and numbers of patients and amount of information on each patient (‘volume’) and are collected for a variety of purposes (such as clinical care, billing, auditing, and quality monitoring).^6–9^

#### Tradeoffs between scale and depth

**Figure 1:**
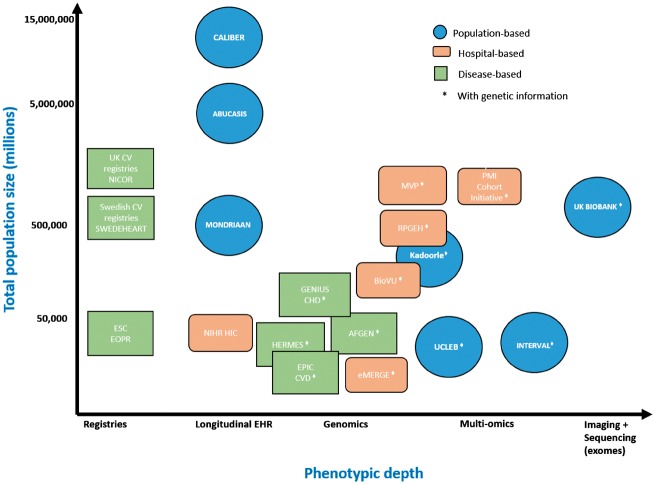
Scale (*N* people), phenotypic and omic resolution of population-based, hospital-based and disease-based exemplar data resources relevant to cardiovascular disease research (for further details see *Boxes 1* and *2*). AF, atrial fibrillation; AFGen, AF Consortium; CHD, coronary heart disease; ESC, European Society of Cardiology; EPIC, European Prospective Investigation into Cancer and Nutrition; ERFC, Emerging Risk Factors Collaboration; eMERGE, Electronic Medical Records and Genomics; HF, heart failure; PMI, precision medicine initiative; MVP, Million Veterans Programme; NICOR, National Institute for Cardiovascular Outcomes Research; NIHR, National Institute for Health Research; RPGEH, Research Programme on Genes, Environment, and Health; UCLEB, University College, London School of Hygiene and Tropical Medicine, Edinburgh, Bristol.

*Figure 1* illustrates the variety and volume of data showing the relation between scale (number of people) and depth of phenotypic and omics information in different settings: national population-based, hospital-based, and disease or procedure based registries. The amount of phenotypic information in hospital EHR is much greater than any single registry; but such deeper hospital EHR data, has been challenging for researchers to access at scale.^10^ Hospital EHR potentially provide phenotypically detailed data on all diseases including clinical blood laboratory values, imaging, clinically used device data, and text.^11–14^ EHR comprise both structured and unstructured electronic data generated and captured during routine clinical care. Structured EHR data are recorded using controlled clinical terminologies [such as Systematized Nomenclature of Medicine - Clinical terms (SMOMED-CT)] or statistical classification systems (such as ICD-9, ICD-9-CM, or ICD-10). Unstructured clinical data such as patient medical histories, discharge summaries, handover notes, and imaging reports are captured and recorded in patient’s health records as raw unformatted text. Such varied data, from different sources, has been likened to a tapestry^15^ which can be woven together using data linkage and integration techniques into a fine-grained longitudinal picture of health over time (the ‘human phenome sequence’). Such diverse data may offer higher resolution of clinically relevant clusters of diseases, causes, and classifiers.

*Figure 1* makes an important distinction between those record resources with, and without genomic information. *Boxes 1* and *2* provide further details of these resources which may be accessed for translational collaborative research. Biobanks and genomics consortia increasingly rely on EHR linkages for the ascertainment, validation, and phenotyping of not only specific disease outcomes but also the entire longitudinal phenome, as captured by an growing array of digital sources.^16^ Thus any one data resource may include combinations of researcher-generated data (such as omics) and researcher-harnessed data from EHR. Recent initiatives, such as the Innovative Medicines Initiative Big Data for Better Outcomes ‘Big Data@Heart’,^17^^,^^18^ and the American Heart Association (AHA) Verily AstraZeneca ‘One Brave Idea’ initiative.^19^ seek to exploit different sources of records and omics data, across multiple consented and anonymized sources—using the human as the ‘new model organism’.

#### Digital trace of health, outwith healthcare

The resources illustrated in *Boxes 1* and *2* are making increasing use of such data sources including the physical environment, consumer information, socioeconomic and behavioural factors^20^^,^^21^ and user-generated data from mobile health apps, wearables, sensors and social media.^22–24^ In particular the ‘always on’ aspects of mobile and wearables provides major opportunities.

In order to exploit these resources for translational research there is an increasing use of computer science approaches to harness publicly available curated knowledge in different fields including: the medical literature (e.g. PubMed), catalogues of genetic variant-phenotype associations (PhenoScanner^25^^,^^26^), disease-agnostic drug targets (e.g. DrugBank^27^), drug compounds (CHEMBL,^28^ and adverse drug reactions (e.g. IMI PROTECT^29^).

#### Volume: scale with cohorts of millions of participants

Higher resolution enquiry of common and rare diseases (or rare outcomes of common diseases, including drug side effects), demands higher sample sizes: 5000 people in the Framingham cohort, 500 000 in UK Biobank,^16^ 15 000 000 in curated, linked EHR cohorts such as CALIBER,^30–32^ (*Figure 3*) and cross-national collections of EHR cohorts in 100 000 000.^33–35^ An individual’s interactions with the healthcare system may also generate big data; in the general population on average one person accumulates 1000 health events over 3 years in national coded data; a single cardiac MR scan has 10^8^ voxels and a clinical grade (×30) whole genome sequence provides 15 Gb of data.^36^^,^^37^

#### Value: opportunity to disrupt current models of research and care

The value of diverse, high volume data is already changing the way that health care is delivered and is yielding insights in early and late translation (see Potential for early translational research section). There are many sources of value in big data, beyond the immediate scientific dimensions of scale and longitudinal phenotypic resolution. These include the *whole-system relevance* when population and healthcare system records are used: for example, in countries with nationwide health record systems, EHR are the only way of obtaining large scale representative samples. The *velocity* of big data is an opportunity for real time analytics with intelligent feedback loops to improve healthcare systems and individual decision making. The exploitation of such rich big record data sources is *more efficient and cost-effective* compared with traditional researcher-led approaches since for example, in EHR cohorts the cost to research funders of baseline and follow up data *collection* is zero (the data exist as part of healthcare systems). The costs however of collating, cleaning and curating these data and meeting the challenges outlined below are substantial and are further elaborated below.

### Big health data challenges

In realizing the opportunities of such diverse, large volume data there are formidable challenges. These include: knowing what data are potentially available, information governance, models of data access (responsible data sharing), building and maintaining public trust, developing standards for defining disease, and developing tools for scalable, replicable science and equipping the clinical and scientific work force with new inter-disciplinary skills.

#### Are the data of sufficient quality for a given research question?

**Figure 2:**
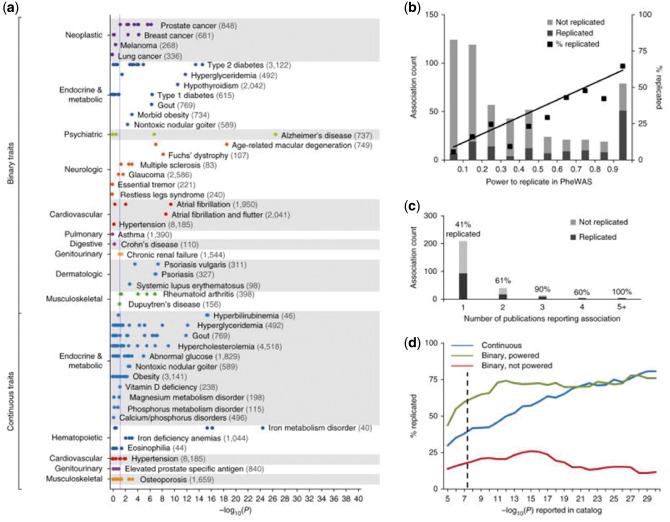
Electronic health record (EHR) Phenome Wide association studies (PheWAS). Source: Denny *et al.*^44^ (reproduced by kind permission). Each point represents the –log_10_(*P*) of a single SNP-phenotype association tested with PheWAS. This study is restricted to SNP-phenotype associations that achieved genome-wide significance (*P* ≤ 5 × 10^−8^) in at least one prior genome wide association study (GWAS) study that included individuals of European ancestry. Numbers in parentheses beside each phenotype represent the sample size within the PheWAS data set. The vertical blue line represents *P* = 0.05. Binary traits refer to all adequately powered, binary traits in the NHGRI Catalog with exact matches to a PheWAS phenotype. For example, 5/5 catalog SNPs associated with rheumatoid arthritis were replicated at *P* < 0.05 in PheWAS, and 9/15 SNPs associated with type 2 diabetes were replicated. Continuous traits are those numerically defined traits in the NHGRI Catalog that are related to PheWAS diseases (e.g. ‘iron deficiency anaemia’ was the PheWAS trait paired with the ‘serum iron level’ catalog trait).

*Challenge*: The quality of EHR data can be said to be ‘in the eye of the researcher’. In any given dataset the amount of missing data, often not missing at random, or inaccurate data, may prohibit valid inference for some but not all research questions. Linked EHR, subject to robust pre-processing and cleaning, have been shown to provide valid measures of risk factors and a wide range of diseases, and therefore offer a common scaffold on which to build specific research questions.^30^*Solution*: A data mantra is ‘collect once, use many times’: and there are calls to make good quality clinical record keeping, as ‘research grade data’. It should be noted that accurate and complete recording, though desirable, does not replace appropriate study design or resolve limitations such as confounding by indication. Validity and data quality may be assessed in multiple ways including:

- *Cross referencing multiple sources of data in the same individuals* (each with their own strengths and limitations): e.g. for acute myocardial infarction linking four national population based sources (primary care, hospital, heart attack and death registries) (CALIBER) shows the positive predictive value and prognostic validity of cases defined in different sources, and allows development and sharing of phenotypic algorithms.^38^^,^^39^ Comparisons of trial adjudicated and medical claims data have been shown to be poor for some endpoints (e.g. bleeding^40^), a comparison of adjudicated endpoints and administrative data showed good agreement.^41^
- *International comparisons*: for example, EHR cohorts in heart attack survivors using ICD codes from different versions (ICD-9-CM, ICD-9, ICD-10) and different countries (US, Sweden, France and England) demonstrated for 12 risk factors consistent relative risks associations with fatal and non-fatal long term outcomes.^42^ In general populations the Emerging Risk Factors Collaboration (ERFC) has shown consistency across continents of risk factor associations with CHD incidence.^43^
- *Genomic approaches* to validating case definitions: across 1000s of hospital ICD codes (‘phenome-wide’), reproduce associations from genome wide association studies obtained one phenotype at a time^25^^,^^44^^,^^45^ (*Table 1*, Denny *et al.*^44^*Figure 2*).

**Table 1:** Early translation exemplars of big health record data research: discovery of disease mechanism, drug development, and precision medicine

Health challenges	Example	Author/year	*N* patients	*N* and type of sources*	Phenotype at baseline	Longitudinal phenotypes, omics and imaging	Analysis approaches
Discovery							
Human knockouts and health	Population based resource for experimental medicine in ‘human knockouts’^62^	Narasimham *et al.* 2016	3.222k	3 Consented cohort Recall by genotype, EHR-1^°,^	Parentally related Pakistani adults recruited from antenatal clinic	Exome sequencing: III rare variant genotype; predicts loss of gene function Result 1358 phenotypes	Genetics Experimental medicine Informatics
Discovery approaches agnostic to disease and biology	GWAS and phenome wide association studies (PheWAS)^44^	Denny *et al.* 2017	13.835k	EHR	1358 phenotypes in Hospital treated patients	Genotyped 3144 SNPs	Informatics GWAS
Discovering new disease sub-types	Heart failure with preserved ejection fraction divided into two groups with differing outcomes^65^	Shah *et al.* 2015	0.397k	5 67 parameters physical characteristics, blood labor, ECG, echo-cardiography	Heart failure (preserved ejection fraction, HFpEF)	HF hospitalization	Machine learning: unbiased hierarchical cluster analysis on continuous values
Developing models of disease networks	Networks of more than 1000 longitudinal disease trajectories: gout important for cardiovascular disease progression^149^	Jensen *et al.* 2014	6.2 m	1 EHR-2^°^ (admissions, outpatients, casualty)	All diagnosed diseases	All diagnosed diseases (14.9 years)	Trajectory/network analysis
Drug discovery and repurposing							
Drug target validation	Inactivating mutation in gene (NPC1L1) mimicking drug (ezetimibe) and effect on LDL cholesterol and coronary disease ^78^	Stitziel *et al.* 2014	7.364k	Various, incl. Biovu & GoDarts	CHD cases	Exon sequencing genetics of NPC1L1	Genetics for drug target validation
Repurposing existing drugs	Mapping GWAS catalogues to druggable genome and 3 tiers of compounds 8 drug target gene associations concordant, 19 discordant^85^ e.g. Tocilizumab licensed for rheumatoid arthritis, being tested for use in coronary disease	Finan *et al.* 2017	>100k in 84 GWAS relevant to CVDs	3 All GWAS, All compounds	84 GWAS in 39 CVDs 388 associations in 670 genes, of which 135 genes drugabble	All compounds with bioactivity against targets 18 844 in CHEMBL Druggable genome	Bioinformatics
Trial endpoint optimization	Heart failure and peripheral arterial disease are comments diseases, seldom prominent in trial endpoints^32^	Rapsomaniki *et al.* 2014	2000k	4 EHR1^°^, QR, A, M	Healthy, free from diagnosed CVDs at baseline	12 incident CVDs over follow up	Cohort epidemiology
Precision medicine							
Pre-emptive pharmacogenomics in care	Genotypes used to select anti-platelet drug, or dosing in warfarin^99^	Van Driest *et al.* 2014	10k	EHR structured and text	Hospital patients at high risk of subsequent receipt of antithrombotics	Clopidogrel CYP2C19; Simva SLC01B1; Warfarin VK0RC1; CYP2C9; thiopurine TPMT; tacrolimus CYP3A5	Demonstration project
Tailoring drug treatment decisions to a patients risk of benefit and harm	Prolonged dual anti-platelet therapy: Development and validation of risk prediction models for benefits (CVD death, MI and stroke) and harms (bleeding) ^100^	Pasea *et al.* 2017	18.307k	4 EHR-1^°^ QR, A, M	Stable CAD 12 months post-AMI	CVD, MI, stroke Bleeding	Multiple prognostic risk models and net benefit

*EHR, electronic health records; QR, quality registry; A, Administrative data; M, mortality; GWAS, genome wide association study.

#### What data exist?

*Challenge*: ‘Genome browsers’ facilitate discovery in biological sciences, but currently the contents of the big data tapestry and whether they are suitable for a particular research purpose are hard to uncover within a researcher's own country, let alone across different countries (see *Figure 1* and *Boxes 1* and *2*). *Solution***:** If big data are to disrupt current research models then there is a need for searchable catalogues of data, metadata, feasibility counts (and ideally sample data) and access arrangements. The creation of public, standards-driven metadata and data portals can assist researchers in locating the right dataset for their research question and obtaining up to date details on data availability and accessibility. For example, the IMI-funded European Medical Information Framework (EMIF) data catalogue contains information on over 300 data sources ranging from EHR, consented cohort studies, and surveillance datasets.

#### What is the legal and ethical framework for using such data?

*Challenge*: The information governance of big health data resources presents major challenges. The need for protecting privacy, confidentiality, discrimination and other potential harms is vital. However how the regulatory environment proportionately balances these concerns with the potential benefits of data sharing (or, indeed, the harms by not sharing) is evolving.

*Solution*: Broad consent models, such as those in UK Biobank, have an important role, recognizing that it is not possible to stipulate all the potential research uses of data, nor how they will change. Some have argued that a new social contract is required with trusted use of data under innovative, proportionate governance delivering benefits to patients and public.^46–48^

#### How are data shared?

*Challenge*: Despite exhortation from funders, journals and the public to share data, all too often this does not happen. Once researchers have permissions to access data, the mode of data sharing may pose challenges to the researcher.

*Solution*: Data sharing may involve: (i) material transfer agreements with data being physically shared e.g. UK Biobank; (ii) role-based secure remote access; (iii) distributed analyses where data remain stored in individual sources. The Global Alliance for Genomics and Health^49^ is establishing a common framework for harmonized sharing of genomic and clinical data. Distributed analytical tools (e.g. DataSHIELD, i2b2) and common data models [e.g. Observational Medical Outcomes Partnership Common Data Model (OMOP CDM)] can facilitate the remote and sequestered processing of complex datasets without the direct need to transfer data directly.

#### How are disease and trait phenotypes defined and shared?

*Challenge*: There is a lack of an international framework for defining, phenotyping, sub-phenotyping and discovering disease phenotypes in the context of health records. There are multiple controlled clinical terminologies and ontologies (including SNOMED-CT, ICD-10, and the Human Phenotype Ontology), but how these terms should be combined to define meaningful entities, let alone how they should be combined with research data is unclear. Currently many diseases lack internationally agreed criteria (preferably in a machine-readable format) for defining cases and non-cases; acute myocardial infarction, type 2 diabetes are exceptions. Current definitions of many diseases such as HF, AF and ACS span heterogeneous groups of patients and describe syndromes only rather than definitions based on understanding of molecular mechanism.

*Solution*: Sharing, validating and refining replicable, scalable EHR phenotypic algorithms requires international efforts.^50^ e.g. PheKB in hospital EHR (codes and text) and national structured records e.g. CALIBER. For example, defining atrial fibrillation using structured national health records may involve several hundred codes for diagnoses, drugs, procedures in a phenotyping algorithm. Clinical information standards such as openEHR^51^ or semantic web technologies^52^^,^^53^ can enable researchers to create computational representations of phenotyping algorithms which facilitate their sharing across the research community.

#### What are the tools, methods and analytic approaches?

*Challenge*: There is a wide array of relevant approaches from quantitative disciplines (mathematics, computer science, statistics, software engineering) and from biological disciplines: until recently these have seldom been focused on big health record data.

*Solution*: While there are 7 million hits per day on the European Bioinformatics Institute website; such national and international resources for health informatics are lacking. There is a need for organizations to be established which provide the analogous reference data, tools and methods in health informatics in general^54^ as well as integration across cardiovascular efforts^55^^,^^56^ in order to scale the science.

#### What skills and training are required?

*Challenge*: Few clinicians and health care professionals have had formal training in informatics, data science, (computer) coding, software development or other increasingly relevant skills. In many countries there are large shortfalls in the number of data scientists that have been trained.

*Solution*: National efforts are likely to be important to substantially increase the number, and change the kind, of people required to deliver data-based medicine: hybrid professionals,(for example sub-specialty physician accreditation in informatics), data scientists, data wranglers, and data-savvy health care professionals.^57^ The 10×10 (‘ten by ten’) program was launched in 2005 by the American Medical Informatics Association (AMIA) and Oregon Health & Science University (OHSU). The genesis for the program came when then-President of AMIA, Dr Charles Safran, called for at least one physician and one nurse in each of the 6000 hospitals in the US to have some training in medical informatics. The National Academy of Science has recommended the importance of agile assembly and rewarding of scientific teams across diverse disciplines including genomics, basic biology, mathematics, computer science, statistics, engineering.

### Potential for early translational research

In this section we provide selected exemplars of the potential of big health record data arising from the variety, volume and value of the data being realized and how big data are contributing to scientific advance in cardiovascular medicine from discovery of underlying disease mechanisms, disease taxonomy, of treatment relevant sub-types of disease which underpin drug development, and precision medicine.^58^^,^^59^

#### Discovery in genetic and EHR data

It is important to note that it is challenging to provide deep mechanistic insight in large scale EHR data resources given the limited availability of genetic information in sufficient depth. Bespoke, recallable investigator-led studies such as East London Genes & Health (ELGH^60^) and the NIHR BioResource^61^ enable the coupling of EHR data with extreme genotypes (or phenotypes) and enable their in-depth study using bespoke experimental protocols.^62^^,^^63^ Complete gene knockouts are highly informative about gene function with a recent study of 3222 British Pakistani-heritage exome-sequenced adults with high parental relatedness, discovered 1111 rare-variant homozygous likely loss of function (rhLOF) genotypes predicted to disrupt (knockout) 781 genes. Linking to EHR, investigators observed no association of rhLOF genotypes with prescription- or doctor-consultation rate, and no disease-related phenotypes in 33 of 42 individuals with rhLOF genotypes in recessive Mendelian disease genes. Phased genome sequencing of a healthy PRDM9 knockout mother, her child and controls, showed meiotic recombination sites localized away from PRDM9-dependent hotspots, demonstrating PRDM9 redundancy in humans. Genomic approaches to validating case definitions: across 1000 s of hospital ICD codes (‘phenome-wide’), reproduce associations from genome wide association studies obtained one phenotype at a time (*Table 1*, Denny *et al.*,^44^*Figure 2*).

#### Discovery in larger scale epidemiology

**Figure 3:**
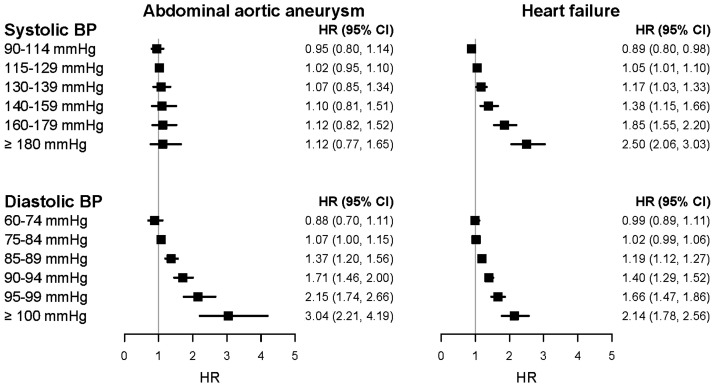
Resolution across a range of risk factor levels (systolic and diastolic blood pressure) and range of different initial presentations of cardiovascular disease (abdominal aortic aneurysm and heart failure only shown here): discovery of heterogeneous associations in a cohort of >1m adults initially free from diagnosed cardiovascular disease using national structured linked electronic health records from the CALIBER resource, in which EHR phenotyping algorithms are created, validated and shared using a robust methodology.^32^^,^^50^

Big health record data can contribute to the discovery of new associations, which would be hard to generate from traditional consented cohorts without record linkage. For example, *Figure 3* and *Table 1*, Rapsomaniki *et al.*^32^ illustrates how the power of large scale health records allows enquiry into less common cardiovascular diseases such as abdominal aortic aneurysm: Here there is a marked discordance between the strong association of diastolic blood pressure with abdominal aortic aneurysm compared with the lack of association with systolic blood pressure. These findings have implications for understanding the aetiology of abdominal aortic aneurysms, screening and prevention and understanding the underlying molecular mechanisms of disease for creating interventions.

A key prerequisite for precision medicine is the estimation of disease progression from the current patient state. Disease correlations and temporal disease progression (trajectories) have mainly been analysed with focus on a small number of diseases or using large-scale approaches without time consideration, exceeding a few years. Investigators performed a discovery-driven analysis of temporal disease progression patterns using data from an electronic health registry covering the whole population of Denmark. Utilizing the entire spectrum of diseases, they convert 14.9 years of registry data on 6.2 million patients into 1171 significant trajectories. Key diagnoses such as gout and chronic obstructive pulmonary disease (COPD) were identified as central to disease progression across many of these trajectories and hence important to diagnose earlier. Such data-driven trajectory analyses may be useful for predicting and preventing future diseases of individual patients.

#### Discovery with deep phenotypic data

Most cardiovascular diseases (including acute myocardial infarction) have syndromic descriptions and labels, which may span multiple underlying pathological disease processes.^64^ One approach to discovering mechanistically relevant disease types is to phenomap disease. For example, *Table 1*, Shah *et al.*,^65^ in heart failure with preserved ejection fraction machine learning on 46 continuous clinical, laboratory, electrocardiographic, and echocardiographic findings has been used to define mutually exclusive groups, which relate to subsequent outcomes.^65–67^ The cardiac atlas project (of normal and diseased hearts) is an example of large scale collaborations on feature extraction in imaging^68^^,^^69^ using data sharing in standard formats Digital Imaging and Communications in Medicine (DICOM) of pixel and non-pixel data.^70^ Personalization using physiological simulations^71^ for example for cardiac resynchronization therapy^71^^,^^72^ is proposed. Unstructured free-text data in EHR may add further resolution for patient stratification and disease co-occurrence estimation, which subsequently can be mapped to systems biology frameworks.^67^

#### Drug development and repurposing

More drugs are required to prevent and treat cardiovascular diseases. Since 2000, the FDA has approved only two new classes of cardiac drugs with widespread application: P2Y12 receptor inhibitors (such as clopidogrel, ticagrelor, prasugrel) and novel oral anti-coagulants (such as dagibatran, apixaban, rivaroxaban, edoxaban). Costly, late drug failures occurring within phase III trials have been recently seen for CETP inhibitors which raise HDL-cholesterol (HDL-C),^73–75^ ivabradine which lowers the heart rate^76^ and darapladib, a selective oral inhibitor of lipoprotein-associated phospholipase A_2_.^77^

#### Discovering and validating drug targets

EHR-DNA resources may play an increasingly important role in drug discovery, genomic drug target validation, marker validation and in drug repurposing. For example, NPC1L1 (*Table 1*, Stitziel *et al*.^78^) demonstrates the strategy that human mutations that inactivate a gene encoding a drug target can mimic the action of an inhibitory drug—here ezetemibe—and thus can be used to infer potential effects of that drug. Ezetemibe is known to affect the marker (LDL cholesterol) but, until recently, not the disease (myocardial infarction). Among the largest sources of cases of MI and controls in this study was a DNA resource integrated into a health system with rich EHR.^78^ The discovery of PCSK9 as a drug target to lower cholesterol,^79^ which could in principle have been made in EHR-DNA resources, illustrates the importance of rare variants in identification of pathways relevant to the whole population. Mendelian randomization studies are important in evaluating whether markers—such as heart rate and HDL cholesterol—are causal for the disease of interest. Such genetic studies have questioned the role of heart rate^80^^,^^81^ and HDL cholesterol^82^ in the aetiology of heart attack.

#### Drug repurposing and PheWAS

Identifying novel disease indications for already approved drugs (repositioning or repurposing) has been successful for sildenafil,^83^ and beta blockers (repurposed for heart failure). The discovery that IL-6 is causally related to myocardial infarction^43^ has led to proposals for repurposing tocilizumab, which is currently licensed for rheumatoid arthritis. Here the question is what other phenotypes are associated with the drug-relevant genetic variant?’ (*Figure 2*) For example, examining 778 disease phenotypes based on ICD codes in the EHR^84^ identified potential novel pleiotropic associations with a variant in the sodium channel gene *SCN10A*. This variant is associated not only with the anticipated arrhythmias, but (possibly) also with unanticipated diseases, here cholecystitis. Recent interest has been to scale this approach to systematically evaluate drugs against a wide range of untested diseases. To be successful this would require substantially larger EHR-DNA resources incorporating longitudinal disease trajectories from big record data^85^ and might aid drug repurposing efforts.

#### Trial endpoint optimization

Drugs may fail in phase III trials because of the composition of primary endpoints. For example, the inclusion of myocardial infarction—which is not causally related to heart rate—in the trial of the heart rate lowering drug ivabradine. In trials of treatments in type 2 diabetes the primary endpoint often includes non-fatal MI, non-fatal stroke and death from cardiovascular diseases. Large scale record cohorts however demonstrate that the initial presentation of cardiovascular disease is commonly heart failure and peripheral arterial disease^86^—neither of which are prominent components of primary trial endpoints. Moreover, inclusion of some diseases might dilute the trial endpoint since type 2 diabetes is associated with a lower risk of aneurysms.^86^ In CALIBER, the ability to reliably resolve 12 different CVDs demonstrates that the majority of incident cases of CVD are neither heart attack nor stroke^86^ and that risk factor associations are heterogeneous across different diseases.^86–89^

#### Trials of new drugs

Once the ‘right drug, the right target and right endpoints’ have been evaluated, the next and most costly hurdle is to carry out the definitive experiment—the phase III trial. Twenty years ago the West of Scotland Coronary Prevention Study (WOSCOPS) statin trial study demonstrated the value of EHR linkage for long-term follow-up of clinical outcomes.^41^^,^^90^ Underpinning regulatory and data standards and interoperability issues^91^ are the focus of international initiatives,^92–94^ but in cardiovascular disease there has not yet been a pragmatic phase III trial of a pre-licence drug. The Salford Lung Study (GSK, relovair) is the world‘s first such trial and is set in a regional ‘whole health system’ EHR.^95^^,^^96^

#### Integrating pharmacogenomics

Multi-scale biological data, when combined with these deeper phenotypes, underpin further dissection of disease. Whole genome sequencing is beginning to be implemented in clinical care, for molecular diagnosis, identification of risk of subsequent wide range of diseases, reproductive considerations and drug response.^36^^,^^97^ It is in drug response that precision medicine is finding early application. Here the goal is to identify biologically relevant subgroups in which either the benefit is greater, or, more commonly, the harms are fewer (interaction on the relative risk scale). Pre-emptive genomic testing, in which actionable genetic variants have already been assessed prior to drug exposure, is beginning to be implemented in the EHR for the care of patients^98^ (*Table 1*, Van Driest *et al.*^99^).

#### Personalized estimates of benefits and harms

One example of the need to individualize risk comes from prolonged dual anti-platelet therapy among patients who have survived 1 year after acute myocardial infarction. For example, *Table 1*, Pasea *et al.*,^100^ in prognostic models for risk of atherothrombotic and bleeding events have recently been developed and validated and allow an updatable estimation of net clinical benefits for each patient to guide the decision for prolonged dual anti-platelet therapy.

Clinical record data are highly effective in distinguishing risk groups, for diverse diseases and in diverse settings^101–103^ and higher risk patients usually have more absolute benefit than those in lower risk groups (i.e. without biologic interaction). Clinical risk prediction algorithms and decision support are rapidly proliferating in CVD and many tools can be envisaged in the management of a single patient, spanning benefits and harms at different time points. Clinical data can outperform the Framingham risk score,^102^ and can flexibly model start point populations and endpoints and be easily updated in the light of new imaging, genetic information, and implemented in clinical practice. Predictions may be improved by incorporating clinical trajectories.^103^ For example patients in whom blood pressure declines over time, without diagnosed heart failure, have a worse survival than those whose blood pressure remains stable.^104^ Using all available data points across data modalities combined with machine learning or Bayesian network models may further add to prediction.^105–107^

### Potential for late translational research

#### Learning health care systems

Increasing costs, complexity of patients and fragmentation of healthcare systems are challenges to delivering high quality care with better outcomes and value. Far from a data-based health care system, all too often there is a largely data free (or data silo‘d) approach where the benefits of science and evidence, and experience of care are characterized by missed opportunities, waste and harm.^108–110^ The state of ‘digital maturity’ in hospitals and health eco-systems, varies hugely. Arguably, more people die from lack of use of data than misuse of any other technology.^111^ The concept of learning health systems puts informatics and big data as a central driver of quality, not only seeking to put what is known to work into practice (closing the ‘second translational gap’) but also contributing in new ways to understanding what is effective.^112–114^ It is worth noting that however ‘big’ the data are observational analyses will not replace the need for randomized intervention studies due to the inherent limitations of observational studies to evaluate reliably any modest effect of interventions.

#### Building trials into health systems

A trial of thrombus aspiration demonstrated the feasibility of randomizing a high proportion of patients at point of care in the setting of a national quality registry^115^^,^^116^ (*Table 2*, Fröbert and James^115^). These findings and the growing evidence that EHR can provide a platform for assessing feasibility, refining protocols and recruiting patients^41^^,^^90^^,^^117^ have stimulated major interest because of the lower cost and higher speed of trial delivery. Pragmatic point-of-care EHR based trials are underway e.g. of high vs. low dose aspirin trial among people with stable coronary disease.^118–120^

**Table 2:** Late translation exemplars of big health record data research: learning health systems, citizen driven health, and public health

Health challenges	Example	Author/year	N patients (000’s)	N and type of sources*	Phenotype at baseline	Longitudinal phenotypes, omics and imaging	Design/Analysis/Disciplines
Learning health systems							
Integrating trials in clinical care	Thrombus aspiration at the time of primary coronary intervention (TASTE trial) has no impact on short or long term outcomes^115^	Fröbert *et al.* 2014	7.244k	3 QR, A, M	STEMI, Angio findings	Follow up for ACM, stent restem, uf MI (1 yr)	RCT: Point of care, registry embedded, pragmatic
Comparing effectiveness of whole health systems	Large differences in care and outcomes between UK and Sweden (all hospitals)^137^	Chung *et al.* 2014	500k	2 QR, M	NSTEMI STEMI	ACM (30 d)	Survival analysis
Vigilance for safety	Mining text could have detected the Vioxx – acute MI signal earlier than conventional pharmacoeopidemology approaches^13^	Lependu *et al.* 2013	1.8k	1 hospital records structured and text	All diagnosed diseases rofecexib	All drug safety signals including (acute MI) 11 m clinical notes	Text mining
Targeting cost effective care	Cost effectiveness decision models provide willingness to pay estimates in different risk groups, and different treatment benefits for stable coronary disease^138^	Asaria *et al.* 2016	100k	4 EHR-primary care, QR, A, M	Stable CAD, stable angina (xxx AMI)	All hospitals, procedures, drug use, resource use	Health economics
Citizen driven health							
Real time real world 24/7 monitoring: the ‘sensed self’	Pacemaker monitoring might lower event rates^141^	Hindricks *et al.* 2014	0.716k	4 Implantable monitor	Heart failure Multi-parameter monitoring	Primary outcome was: ACM, hospitalization for heart failure, worsening of NYHA class.	RCT of a detailed monitoring intervention
Delivering individualized interventions through mobile phones	Texts might increase smoking cessation^142^	Free *et al.* 2011	5.8k		Smoker	Cessation Text messaging	RCT of behavioural intervention
Understanding the public through social media	Twitter language might predict community heart disease rates^23^	Eichstaedt *et al.* 2015	148 m country mapped	CDC atherosclerosis Twitter	Mapping of words used in tweets to psychological constructs	N/A	Ecological correlations at county level
Public health							
Epidemiology of all CVDs and clinically relevant sub-types of disease	Incidence and survival of NSTEMI and STEM ICD9-CM^145^	Yeh *et al.* 2010	3000k	2 HMO, M	46 086 hospitalizations STEMI/NSTEMI	All cause mortality30 day	Cohort
Rare disease epidemiology	Rare disease: valid EHR phenotypes & new associations with coronary disease [HCM]^151^	Pujades-Rodriguez *et al.* 2016	1.16k	4 EHR-primary care A, QR, M	Hypertrophic cardiomyopathy	Coronary, stroke, HF, arrhythmia, bleeding, DVT/PE at 4 years follow up	Cohort
Evaluating population impact of interventions	Introduction of smoke free legislation in different countries at different times: impact on admissions to hospital with heart attack England smoke-free 1 July 2007^160^	Sims *et al.* 2010	millions	1 HES	MI admission 1 July 2002–30 September 2008	N/A	Natural experiment Time series analysis

EHR, electronic health records; QR, quality registry; A, Administrative data; M, mortality; GWAS, genome wide association study; HCM, Hypertrophic Cardiomyopathy; MI, myocardial infarction; STEMI, ST-segment elevation MI; NSTEMI, Non ST-segment elevation MI; CAD, Coronary Artery Disease; ACM, All-Cause Mortality; RCT, Randomized Clinical Trial; NYHA, New York Heart Association; ICD9-CM, International Classification of Diseases 9th revision – Clinical Modifications; CVD, Cardiovascular Disease; HMO, Health Management Organisation; CDC, Centres for Disease Control and Prevention; HES, Hospital Episode Statistics; DVT, Deep Vein Thrombosis; PE, Pulmonary Embolism.

#### Building quality into healthcare delivery: decision support and data based medicine

Early examples of data-based medicine are already here, with clinical data providing both the ‘brain’ to understand what needs fixing and the ‘spinal cord’ to help fix it. For example, analysis of health record cohorts provides understanding of the patient journey and cumulative missed opportunities of cardiovascular care over time^121^^,^^122^ and may provide risk prediction tools which are derived from clinical data, and used in practice to support healthcare decision making.^102^^,^^123^

A small but growing number of hospitals have a suite of readily modifiable information feedback loops to improve care.^124^ There is a need for more empirical demonstration of the impact on outcomes of these systems. A key challenge lies in intelligent real time systems.^125–127^ Practice-based medicine^128^^,^^129^ involves large-scale, real time studies (based on a health system‘s own data) to generate evidence directly relevant to the patient in front of the clinician. Sometimes this observation is sufficient, sometimes it allows systematic identification of the need for trials. These trials may exploit the efficiency of big data in point-of-care individual patient randomized trials embedded in a learning health system or may involve randomizing clusters of health care professionals, for example to evaluate complex interventions, such as decision support.^130^^,^^131^

#### Big data for safety vigilance

Mining EHR in real time with both coded and text data is an important source of safety information. For example, *Table 2*, Lependu *et al.*,^13^ the excess myocardial infarction risk associated with rofecoxib (Vioxx) could have been detected 1–2 years earlier had records. There are international initiatives to achieve the vast scale required to evaluate drug safety in up to 150 million patients.^34^^,^^132–135^ Using the Medicare Patient Safety Monitoring System there was a decline in adverse events following heart attack and heart failure, but not for pneumonia or conditions requiring surgery,^136^ possibly as a result of more organized quality initiatives in the cardiovascular diseases.

#### International comparisons of whole system care and outcomes

Nationwide, policy relevant comparisons of care and outcomes among people with CVDs across health systems have only recently been reported. For example, *Table 2*, Chung *et al.*,^137^ using data from ongoing quality registries from all hospitals in Sweden and the UK, including more than half a million patients, demonstrates that 30-day MI mortality was higher in the UK than in Sweden. Politicians, policy makers and health care professionals seek to make claims that their health systems deliver world class care and outcomes—ongoing, even semi-automated comparisons across countries might be used to evaluate whether such claims are ‘data-based’.

#### Cost effectiveness of innovation

Big data provide new opportunities in understanding the cost effectiveness of existing and new interventions. Because of the ability to assess baseline risks in unselected general populations (commonly higher risk than those reported in trials), such ‘real world evidence’ is increasingly required by payers and the regulators. As more data sources are linked, greater granularity of the care data (e.g. 67 different types of primary care ‘consultation’) may provide more accurate and more complete resource use data. For example, *Table 2*, Asaria *et al*.,^138^ cost-effectiveness decision models can be developed before trials report to estimate the willingness to pay and pricing of a drug according to different trial benefits (relative risk reductions) applied to patients at different strata of risk.

#### Citizen-centred health

People increasingly have more and different information than their doctor or researcher raising new possibilities of ‘disintermediation’, potentially disrupting current models of health care and research.^139^

The heart and circulation are increasingly observable as a ‘sensed self’ with novel wireless devices for mobile monitoring, with huge new data streams.^22^^,^^140^ Smartphone apps and sensors are available to record and transmit to physician, electrocardiograms (e.g. to screen for atrial fibrillation), heart rate, blood pressure, radial artery waveforms, respiratory rate, oxygen saturation, temperature, even ultrasound.^22^ These may provide deeper, naturalistic phenotyping in areas often lacking in the clinical record, including: physical activity, weight, diet, sleep, quality of life, and symptoms and medication compliance. For accelerometry questions remain about how best to analyse and present such data.

Implantable devices such as pacemakers provide tele-monitoring data which might reduce the risk of fatal and non-fatal outcomes in patients with heart failure (*Table 2*, Hindricks *et al.*^141^). Interventions can be delivered through mobile means and text messaging may increase smoking cessation rates (*Table 2*, Free *et al.*^142^). Apple ResearchKit provides new ways to recruit people rapidly into studies.

Open, publicly available data donated and shared by citizens is becoming increasingly available. User generated content in social media are inherently public and the language used in twitter can be used to predict community heart disease rates (*Table 2*, Eichstaedt *et al.*^23^) and it is plausible that Google searches^24^ might give clues to environmental pollution triggers of acute cardiovascular events. As patients increasingly access, own and control their health records^143^ they may share their clinical records, genetic and other data through initiatives like ‘Patients like me’ and ‘23 And Me’, offering networks of individuals to develop communities of interest e.g. in rare diseases for orphan drugs. Citizens may do their own science; with schoolchildren exploiting publically available data to develop diagnostic tools using artificial neural networks.^144^

#### Public health

There are major gaps in our ability to prevent the onset of and prolong life in, many of the most common cardiovascular diseases in the 21st century including atrial fibrillation, heart failure, peripheral arterial disease. There are also gaps in our ability to measure disease and model the impact of interventions in populations. Clinicians diagnose more specific entities than ‘heart attack’, ‘CHD’ or ‘CVD’ yet conventional consented cohorts have lacked the statistical size or the phenotypic resolution to measure clinically relevant sub-types of disease. Big data can study the diseases that clinicians diagnose to provide scalable, population based, updatable measurements of modern disease burden vital for the evaluation of alternative strategies of prevention. For example, big data can be used to estimate the incidence and survival of the treatment-relevant sub-types of MI (ST elevation and non-ST elevation) (*Table 2*, Exemplar Yeh *et al.*^145^ or stable angina).^146^

#### Meaningfully complex models of public health

Existing models of disease prevention are simple and often focus on one disease or one risk factor at a time. Big data invite a richer understanding of the importance of: multiple diseases co-occurring^147^; networks of risk factors (obesity^20^ and smoking^148^ and diseases^149^; fine-grained geospatial resolution; rare^150^*Table 2*, Pujades-Rodriguez *et al.*^151^ and common diseases; diseases as causes or triggers of cardiovascular events^152^; diseases of developing^101^ and developed countries, and across multiple biological scales through to societal influences on health). In order to understand weather and climate big data, with appropriately complex mathematical models, are used in national institutes,^153^ but no such analogue exists for public health.

#### Big socio-economic data

Unlike many technological advances, big data may have a role in actionable understanding of, and reductions in, inequalities in health and healthcare in rich and poor countries. The opportunity to move to a neighbourhood with lower poverty may reduce obesity and diabetes.^154^ The data in this trial were collected through traditional means, but such data could have been captured in part with cross-government record linkages. Big data are important for achieving sustainable development goals^155^ and recommendations have been made for the recording social and behavioural determinants in the clinical record.^156^ Linking health record data to an individual‘s lifelong tax contributions may provide new policy relevant insights into the relations between wealth and health.^157^^,^^158^ Cross-government approaches to big data might open up enquiry into neglected populations with insights to improve the cardiovascular health of those on social welfare benefits, the homeless, refugee, and prison populations.

#### Population impact of interventions

Big data can be used to evaluate the population impact of healthcare or public health interventions.^159^ For example, *Table 2*, Sims *et al.*,^160^ shows how health records have been used to demonstrate the impact of the public smoking ban on hospital admissions for heart attack.^160–162^ Importantly, big health data are a means to evaluate the impact on population health of primary care^163^ the state of digital maturity of a hospital or health system^164^ or the existence of quality and outcome registries.

## Conclusion

Big health record data are beginning to disrupt the nature of cardiovascular research as well as models of care. Exploiting such data is beginning to improve understanding of cardiovascular disease causation and classification, and contributing actionable analytics to improve health and healthcare, but major challenges need to be addressed to realize more fully their potential.
